# Maternal and neonatal risk factors for autism spectrum disorder: A case-control study from Egypt

**DOI:** 10.1371/journal.pone.0269803

**Published:** 2022-06-15

**Authors:** Ahmed Arafa, Omaima Mahmoud, Hisham Salah, Ahmed Ali Abdelmonem, Shaimaa Senosy

**Affiliations:** 1 Department of Public Health, Faculty of Medicine, Beni-Suef University, Beni-Suef, Egypt; 2 Department of Psychiatric Nursing, Faculty of Nursing, Beni-Suef University, Beni-Suef, Egypt; 3 Department of Psychiatry, Faculty of Medicine, Beni-Suef University, Beni-Suef, Egypt; 4 Unit of Phoniatrics, Department of Otolaryngology, Faculty of Medicine, Beni-Suef University, Beni-Suef, Egypt; Chinese Academy of Medical Sciences and Peking Union Medical College, CHINA

## Abstract

**Background:**

The prevalence of autism spectrum disorder (ASD) has been increasing steadily in Egypt and worldwide. Detecting risk factors for ASD could help initiate screening and risk prevention approaches. Herein, this study aimed to detect several maternal and neonatal risk factors for ASD in Egypt.

**Methods:**

In this case-control study, mothers of children with ASD who were visiting Beni-Suef University Hospital in Egypt (n = 268) were compared to mothers of children without ASD attending one primary school with a kindergarten (n = 504) regarding their preconception, conception, and postconception characteristics. Data were collected using a self-administered questionnaire. The odds ratios (ORs) with 95% confidence intervals (CIs) were calculated to investigate the possible associations between the collected data and the odds of ASD.

**Results:**

In the multivariable-adjusted models, urban residence: OR (95% CI) = 2.33 (1.60–3.38), relative father: 2.63 (1.74–3.96), history of diabetes: 5.98 (1.99–17.97), previous abortion: 2.47 (1.20–13.38), assisted fertility: 4.01 (1.20–13.38), family history of ASD: 7.24 (2.00–26.24), multiple pregnancy: 11.60 (2.54–53.07), exposure to passive smoking during pregnancy: 2.95 (1.86–4.68), vaginal bleeding during pregnancy: 3.10 (1.44–6.67), hypertension with pregnancy: 3.64 (1.06–12.51), preterm labor: 2.64 (1.26–5.57), neonatal convulsions: 14.88 (5.01–44.20), and admission to neonatal intensive care unit 2.13: (1.21–3.74) were associated with the increased odds of ASD. On the other hand, the intake of vitamins during pregnancy: 0.09 (0.06–0.16) and C-section: 0.44 (0.27–0.70) were associated with the decreased odds of ASD.

**Conclusion:**

This study detected several maternal and neonatal risk factors for ASD in Egyptian children.

## 1. Introduction

Autism spectrum disorder (ASD) is a term referring to a constellation of early-appearing deficits in social, emotional, and nonverbal communications in addition to strict or repetitive behaviors [1, 2]. The disorder, which has a global prevalence of 0.5% to 2% [3], results in a substantial social and economic burden [4, 5]. Since early diagnosis and behavioral intervention in ASD could effectively improve prognosis, detecting ASD risk factors to identify at-risk children should be encouraged [6].

While ASD is strictly linked to genetic factors [7–10], cumulating epidemiological evidence has suggested that maternal and neonatal factors could play important roles as well [11–30]. Yet, the majority of the epidemiological studies were conducted on Western populations [19–30]. Besides, a previous multi-ethnic study found that risk factors for ASD varied across races [23]. Another study detected noticeable differences in ASD risk factors across five countries [24]. Therefore, applying the findings of the Western studies to non-Western populations would be inaccurate. Further, most epidemiological studies assessed selected lifestyle or environmental factors and even reached inconsistent findings [19–30].

Egypt, on the other hand, has one of the highest fertility and population growth rates worldwide; 3.3 and 2.1%, respectively [31]; therefore, it can be speculated that the number of ASD patients in the country has been increasing accordingly. However, no previous studies have comprehensively studied the risk factors for ASD in Egypt.

In this context, we conducted a case-control study to investigate the potential associations of several maternal and neonatal risk factors with ASD using a sample from Egypt.

## 2. Methods

### 2.1. Study population

In this case-control study, mothers of children with ASD were compared to mothers of children without ASD. During the period between July 2019 and January 2021, mothers of children with ASD were recruited from Beni-Suef University Hospital where their children were receiving psychiatric, speech, phoniatric, and dietetic consultations. Beni-Suef University Hospital is a teaching hospital that offers primary, secondary, and tertiary healthcare services to the residents of Beni-Suef Governorate in South Egypt.

Out of 308 invited mothers of children with ASD, 268 accepted to participate with a response rate of 87%. To recruit mothers of children without ASD to serve as controls, invitation letters were sent in March 2020 to the mothers of children attending one public primary school with a kindergarten close to Beni-Suef University Hospital. Mothers who gave their written approvals received the study questionnaire. To minimize the potential impact of recall bias, we asked the participating mothers from the control group to report the data of the youngest living child only. Eventually, out of 1018 mothers invited, 718 gave their acceptance with an acceptance rate of 70.5%. Out of 718 questionnaires sent, 545 were sent back with responses, yet we removed 41 questionnaires for incomplete data (n = 38) or having other children with ASD (n = 3). No incentives were offered for participation.

### 2.2. ASD ascertainment

ASD, in this study, was diagnosed by psychiatric specialists, according to the American Psychiatric Association’s Diagnostic and Statistical Manual, Fifth Edition (DSM-5), when children had persistent deficits in each of the following communications: 1) social-emotional reciprocity, 2) nonverbal communicative behaviors, and 3) developing and understanding relationships in addition to at least two of the following types: 1) stereotyped movements or using of objects, 2) insistence on sameness, 3) restricted interests, and 4) hypo- or hyperreactivity to sensory inputs [1].

### 2.3. Assessment of risk factors

Data on risk factors were collected from mothers of children with and without ASD using a self-administered questionnaire in the Arabic language. The questionnaire was designed by the authors of this study after revising the literature and was composed of four sections. Section I included information about the child: name, age (number in years), sex (male or female), order (number), and age of ASD diagnosis (number in years) (in the ASD questionnaires only). Section II included data about the mother before conception: residence (urban or rural), job (yes or no), education (none, elementary or preparatory, high school or equal, or university and higher studies), the father of the child is a relative (yes or no), histories of hypertension, diabetes, bronchial asthma, and psychiatric diseases (yes or no, each), family history of ASD (yes or no), using assisted fertility (yes or no), and previous abortion (yes or no). Section III included data about the mother during conception: age at pregnancy (years), multiple pregnancy (yes or no), exposure to passive smoking and pesticides (yes or no, each), intakes of antibiotics and vitamins (yes or no, each), history of vaginal bleeding during pregnancy, diabetes with pregnancy, hypertension with pregnancy, and preterm labor (yes or no, each), and mode of delivery (normal or C-section). Section IV included data about the child after conception: low birthweight (yes or no), neonatal respiratory distress and convulsion (yes or no, each), and admission to neonatal intensive care unit (yes or no). Mothers who could not read or write and those who were not able to understand certain questions were asked to seek help from the study investigators who made their contact details available. It took most mothers 15–25 minutes to complete the questionnaire.

### 2.4. Statistical analysis

The logistic regression analyses were used to calculate the odds ratios (ORs) and their 95% confidence intervals (95% CIs) for maternal and neonatal risk factors among mothers of children with ASD compared with mothers of children without ASD. All associations were presented unadjusted (model I) and adjusted for sex and order of the child and preconception variables (model II). In addition to the first two models, every conception variable was adjusted further for other conception variables (model III), and every postconception variable was adjusted further for other postconception variables (model IV). Data were analyzed using the Statistical Package for Social Science (SPSS) released in 2013 (IBM SPSS Statistics for Windows, Version 22.0, IBM Corporation, Armonk, New York).

### 2.5. Ethical considerations

The Research Ethics Committee of the Faculty of Medicine, Beni-Suef University approved the study protocol. We conducted the study in full accordance with the guidelines for Good Clinical Practice and the Declaration of Helsinki. The details, eligibility criteria, and aims of this study were described for the included mothers who signed their written informed consent forms before participation.

## 3. Results

This study included mothers of 268 children with ASD (70.1% males, 29.9% females) and mothers of 504 children free of ASD (44.0% males and 56.0% females). The mean age of children with ASD was 8.82±3.47 years and that of the children without ASD was 6.42±1.95 years, and the mean age of ASD diagnosis was 4.68±2.03 years. In general, children in the ASD group were more likely to be males and the eldest of their siblings (p-value< 0.001) (Table 1).

**Table 1:** Characteristics of children.

Characteristics		Cases	Controls	P-value
Frequency		268	504	--
Sex, %	Male	70.1	44.0	<0.001
	Female	29.9	56.0	
Order, %	Eldest	47.0	23.2	<0.001
	Younger	53.0	76.8	
Current age in years, mean ± Sd (range)		8.82±3.47 (2–13)	6.42±1.95 (3–12)	<0.001
Age at diagnosis in years, mean ± Sd (range)		4.68±2.03 (1–9)	--	--

Regarding the preconception variables, urban residence: OR (95% CI): 2.96 (2.17–4.02), higher education: 1.37 (1.02–1.85), relative father: 2.81 (1.99–3.98), positive histories of hypertension: 2.18 (1.13–4.19), diabetes: 6.76 (2.47–18.53), and bronchial asthma: 2.06 (1.09–3.87), previous abortion: 3.81 (2.57–5.66), assisted fertility: 9.54 (3.21–28.34), and family history of ASD: 14.20 (4.20–48.06) were associated with the increased odds of ASD in the unadjusted model. After adjusting for sex and order of the child and other preconception variables, urban residence: 2.33 (1.60–3.38), relative father: 2.63 (1.74–3.96), positive history of diabetes: 5.98 (1.99–17.97), previous abortion: 2.47 (1.56–3.91), assisted fertility: 4.01 (1.20–13.38), and family history of ASD: 7.24 (2.00–26.24) remained statistically significant (Table 2).

**Table 2:** Preconception maternal predictors of autism spectrum disorder.

Characteristics		Cases %	Controls %	Model I	Model II
Residence	Rural	38.1	64.5	1	1
	Urban	61.9	35.5	2.96 (2.17–4.02)	2.33 (1.60–3.38)
Job	None	66.4	69.8	1	1
	Yes	33.6	30.2	1.17 (0.85–1.61)	0.75 (0.50–1.13)
Education	None or basic	41.0	48.8	1	1
	High or university	59.0	51.2	1.37 (1.02–1.85)	1.03 (0.70–1.53)
The father of the child is a relative	No	65.7	84.3	1	1
	Yes	34.3	15.7	2.81 (1.99–3.98)	2.63 (1.74–3.96)
History of hypertension	No	92.5	96.4	1	1
	Yes	7.5	3.6	2.18 (1.13–4.19)	1.66 (0.76–3.62)
History of diabetes	No	93.7	99.0	1	1
	Yes	6.3	1.0	6.76 (2.47–18.53)	5.98 (1.99–17.97)
History of bronchial asthma	No	92.2	96.0	1	1
	Yes	7.8	4.0	2.06 (1.09–3.87)	2.15 (0.99–4.68)
History of psychiatric disorders	No	95.9	97.6	1	1
	Yes	4.1	2.4	1.76 (0.76–4.03)	1.44 (0.54–3.85)
Previous abortion	No	70.9	90.3	1	1
	Yes	29.1	9.7	3.81 (2.57–5.66)	2.47 (1.56–3.91)
Assisted fertility	No	92.9	99.2	1	1
	Yes	7.1	0.8	9.54 (3.21–28.34)	4.01 (1.20–13.38)
Family history of autism spectrum disorder	No	92.2	99.4	1	1
	Yes	7.8	0.6	14.20 (4.20–48.06)	7.24 (2.00–26.24)

Model I: Unadjusted

Model II: Adjusted for sex and order of the child and other preconception variables

During conception, multiple pregnancy: 14.20 (4.20–48.06), exposure to passive smoking: 2.88 (2.11–3.93) and pesticides: 2.91 (1.17–7.20), vaginal bleeding during pregnancy: 6.19 (3.53–10.86), diabetes with pregnancy: 4.68 (2.11–10.36), hypertension with pregnancy: 3.62 (1.43–9.19), and preterm labor: 2.85 (1.68–4.84) were associated with the increased odds of ASD while vitamin intake: 0.10 (0.07–0.15) was associated with the decreased odds of ASD in the unadjusted model. After adjusting for sex and order of the child, preconception variables, and other conception variables, the following variables predicted ASD: multiple pregnancy: 11.60 (2.54–53.07), exposure to passive smoking: 2.95 (1.86–4.68), threatened abortion: 3.10 (1.44–6.67), hypertension with pregnancy: 3.64 (1.06–12.51), and preterm labor: 2.64 (1.26–5.57) while intake of vitamins: 0.09 (0.06–0.16) and C-section: 0.44 (0.27–0.70) were associated with lower odds of ASD (Table 3).

**Table 3:** Conception maternal predictors of autism spectrum disorder.

Characteristics		Cases %	Controls %	Model I	Model II	Model III
Age at pregnancy	<35 years	85.1	89.3	1	1	1
	≥35 years	14.9	10.7	1.46 (0.94–2.27)	1.60 (0.95–2.72)	1.48 (0.78–2.80)
Multiple pregnancy	No	92.2	99.4	1	1	1
	Yes	7.8	0.6	14.20 (4.20–48.06)	11.49 (2.94–45.02)	11.60 (2.54–53.07)
Exposure to passive smoking	No	50.0	74.2	1	1	1
	Yes	50.0	25.8	2.88 (2.11–3.93)	3.16 (2.16–4.61)	2.95 (1.86–4.68)
Exposure to pesticides	No	95.5	98.4	1	1	1
	Yes	4.5	1.6	2.91 (1.17–7.20)	3.44 (1.27–9.37)	2.72 (0.70–10.56)
Intake of antibiotics	No	78.7	81.3	1	1	1
	Yes	21.3	18.7	1.18 (0.82–1.70)	0.77 (0.49–1.20)	0.70 (0.41–1.20)
Intake of vitamins	No	48.1	8.3	1	1	1
	Yes	51.9	91.7	0.10 (0.07–0.15)	0.07 (0.04–0.12)	0.09 (0.06–0.16)
Vaginal bleeding	No	81.3	96.4	1	1	1
	Yes	18.7	3.6	6.19 (3.53–10.86)	4.58 (2.41–8.67)	3.10 (1.44–6.67)
Diabetes with pregnancy	No	92.2	98.2	1	1	1
	Yes	7.8	1.8	4.68 (2.11–10.36)	2.88 (1.15–7.24)	1.75 (0.57–5.39)
Hypertension with pregnancy	No	95.1	98.6	1	1	1
	Yes	4.9	1.4	3.62 (1.43–9.19)	3.31 (1.11–9.85)	3.64 (1.06–12.51)
Preterm labor	No	86.6	94.8	1		
	Yes	13.4	5.2	2.85 (1.68–4.84)	2.83 (1.54–5.17)	2.64 (1.26–5.57)
Mode of delivery	Normal	31.0	26.8	1	1	1
	C-section	69.0	73.2	0.82 (0.59–1.13)	0.57 (0.39–0.84)	0.44 (0.27–0.70)

Model I: Unadjusted

Model II: Adjusted for sex and order of the child and preconception variables

Model III: Adjusted for model II and other conception variables

After conception, neonatal respiratory distress: 1.95 (1.20–3.19), neonatal convulsions: 9.97 (4.35–22.87), and admissions to neonatal intensive care unit: 4.13 (2.90–5.87) were associated with the increased odds of ASD in the unadjusted model, yet the association of neonatal respiratory distress with ASD disappeared in the adjusted models (Table 4).

**Table 4:** Postconception neonatal predictors of autism spectrum disorder.

Characteristics		Cases %	Controls %	Model I	Model II	Model III	Model IV
Low birthweight	No	78.4	80.4	1	1	1	1
	Yes	21.6	19.6	1.13 (0.79–1.63)	0.95 (0.61–1.47)	0.87 (0.50–1.50)	0.82 (0.46–1.44)
Respiratory distress	No	86.9	92.9	1	1	1	1
	Yes	13.1	7.1	1.95 (1.20–3.19)	1.41 (0.80–2.50)	1.02 (0.50–2.11)	0.77 (0.36–1.66)
Neonatal convulsions	No	87.7	98.6	1	1	1	1
	Yes	12.3	1.4	9.97 (4.35–22.87)	8.21 (3.23–20.90)	14.45 (4.83–43.29)	14.88 (5.01–44.20)
Admission to neonatal intensive care unit	No	60.4	86.3	1	1	1	1
	Yes	39.6	13.7	4.13 (2.90–5.87)	2.99 (1.98–4.53)	1.95 (1.14–3.34)	2.13 (1.21–3.74)

Model I: Unadjusted

Model II: Adjusted for sex and order of the child and preconception variables

Model III: Adjusted for model II and conception variables

Model IV: Adjusted for model III and postconception variables

## 4. Discussion

To the best of our knowledge, this is the first study to examine several maternal and neonatal risk factors for ASD in Egypt. We detected that urban residence, relative father, history of diabetes, previous abortion, assisted fertility, family history of ASD, multiple pregnancy, exposure to passive smoking during pregnancy, vaginal bleeding during pregnancy, hypertension with pregnancy, preterm labor, neonatal convulsions, and admission to neonatal intensive care unit were associated with the increased odds of ASD while intakes of vitamins during pregnancy and C-section were associated with the decreased odds of ASD.

Our findings came in line with the literature. A previous study using Australian data showed that multiple pregnancy and preterm labor were associated with significant increases in the odds of ASD [19]. Another study using a Canadian database detected that maternal history of psychiatric or neurological disorders was associated with a higher risk of ASD [21]. A large retrospective cohort study of 594,638 children from California showed that several perinatal, antepartum, and intrapartum conditions could be associated with the risk of ASD such as hypertension with pregnancy, placental abruption, fetal dystocia, prolapsed cord, birth asphyxia, and the low Apgar score [23].

It was also obvious, in our study, that male sex and being the first child were associated with ASD which agreed with most previous literature [22]; therefore, we controlled for both factors in all regression models to verify that the associations between the investigated preconception, conception, and postconception factors with ASD were independent of the sex of the child and his/her order. However, it should be noted that the association of ASD with birth order may be due to the study design (selection criteria for controls).

Of note, our manuscript studied several maternal and neonatal risk factors for ASD among a non-Western population and used a standardized method for ASD diagnosis; however, several limitations should be considered. First, the retrospective design of this study cannot imply causality; therefore, future prospective studies are needed to confirm our findings. Second, data on maternal and neonatal variables were collected using a self-administrated questionnaire and were not linked to hospital records; consequently, the possibility of recall, information, and misclassification forms of bias is likely. For the same reasons, it was difficult to assess many conception and postconception conditions such as weight gain during pregnancy, placental and umbilical cord complications, and the Apgar score of the infants. Third, it could be speculated that our sample was biased towards severe ASD because we included mothers of children who were receiving medical consultations for different conditions related to ASD. Other less severe ASD cases might have been underrepresented. Fourth, we cannot exclude the possibility that the children of mothers in the control group might have undiagnosed children with ASD because we did not screen their children for ASD. Fifth, we had no data about the developmental and psychiatric disorders that can accompany ASD such as intellectual disability, developmental dyslexia, and depression. However, a previous study showed no differences in the risk factors for ASD between ASD children with and without comorbidities [23]. Sixth, some reported associations with ASD, particularly those that would rely on medical diagnoses, such as the history of hypertension, diabetes, asthma, and psychiatric disorder, could be attributed to higher access to medical care in mothers with ASD children. Seventh, since this study had an observational design, residual confounding could not be excluded.

In conclusion, we detected several maternal and neonatal risk factors for ASD in a cohort from Egypt. We believe that the findings of this study can help in future national screenings and educational programs. Longitudinal studies that link maternal and neonatal data of Egyptian mothers and their offspring to hospital records are warranted. Our study raised the potential for early identification of at-risk children who can benefit from interventions.

## Supporting information

S1 File SPSS file of the raw data. (SAV)
